# Structure and Biosynthesis of Two Exopolysaccharides Produced by *Lactobacillus johnsonii* FI9785*

**DOI:** 10.1074/jbc.M113.507418

**Published:** 2013-09-09

**Authors:** Enes Dertli, Ian J. Colquhoun, A. Patrick Gunning, Roy J. Bongaerts, Gwénaëlle Le Gall, Boyan B. Bonev, Melinda J. Mayer, Arjan Narbad

**Affiliations:** From the ‡ Gut Health and Food Safety Programme, Institute of Food Research, Colney, Norwich NR4 7UA, United Kingdom,; the § Department of Food Engineering, Faculty of Engineering, Bayburt University, Bayburt 69000, Turkey, and; the ¶ Analytical Sciences Unit, Institute of Food Research,; the ‖ Food and Health Programme, Institute of Food Research, and; the ** School of Biomedical Sciences, University of Nottingham, Nottingham NG7 2UH, United Kingdom

**Keywords:** Atomic Force Microscopy, Bacteria, Carbohydrate Structure, Mutant, Nuclear Magnetic Resonance, Lactobacillus johnsonii, eps Cluster, Exopolysaccharide

## Abstract

Exopolysaccharides were isolated and purified from *Lactobacillus johnsonii* FI9785, which has previously been shown to act as a competitive exclusion agent to control *Clostridium perfringens* in poultry. Structural analysis by NMR spectroscopy revealed that *L. johnsonii* FI9785 can produce two types of exopolysaccharide: EPS-1 is a branched dextran with the unusual feature that every backbone residue is substituted with a 2-linked glucose unit, and EPS-2 was shown to have a repeating unit with the following structure: -6)-α-Glc*p*-(1–3)-β-Glc*p*-(1–5)-β-Gal*f*-(1–6)-α-Glc*p*-(1–4)-β-Gal*p*-(1–4)-β-Glc*p*-(1-. Sites on both polysaccharides were partially occupied by substituent groups: 1-phosphoglycerol and *O*-acetyl groups in EPS-1 and a single *O*-acetyl group in EPS-2. Analysis of a deletion mutant (Δ*epsE*) lacking the putative priming glycosyltransferase gene located within a predicted *eps* gene cluster revealed that the mutant could produce EPS-1 but not EPS-2, indicating that *epsE* is essential for the biosynthesis of EPS-2. Atomic force microscopy confirmed the localization of galactose residues on the exterior of wild type cells and their absence in the Δ*epsE* mutant. EPS2 was found to adopt a random coil structural conformation. Deletion of the entire 14-kb *eps* cluster resulted in an acapsular mutant phenotype that was not able to produce either EPS-2 or EPS-1. Alterations in the cell surface properties of the EPS-specific mutants were demonstrated by differences in binding of an anti-wild type *L. johnsonii* antibody. These findings provide insights into the biosynthesis and structures of novel exopolysaccharides produced by *L. johnsonii* FI9785, which are likely to play an important role in biofilm formation, protection against harsh environment of the gut, and colonization of the host.

## Teaser

**Background:** Bacterial cell surface polysaccharides are important in pathogenesis, cell adhesion, and protection against harsh environments.

**Results:** Two novel exopolysaccharide (EPS) structures were identified in *Lactobacillus johnsonii*.

**Conclusion:** The *eps* cluster is essential for production of both EPS, but *epsE* is required only for the heteropolymer.

**Significance:** This study will guide functional analysis of EPS in survival and colonization of gut commensals.

## Introduction

Exopolysaccharides (EPS)^4^ encapsulate some bacteria, either remaining bound to the cell or being released into the environment (1, 2). They have been shown to be important for the genus *Lactobacillus* for their putative roles in colonization, adhesion, stress resistance, host-bacteria interactions, and also immunomodulation, which are all important properties related to their probiotic functions (3). EPS are also of considerable interest to the food industry, due to their rheological properties and GRAS (generally regarded as safe) status (1). The structure of bacterial EPS has a wide diversity among different species due to the different sugar monomers and glycosidic linkages present in their repeating units. Those containing only one type of sugar molecule are described as homopolysaccharides, whereas heteropolysaccharides are composed of different sugar monomers (2, 3). The structural differences of the capsular EPS influence their functional characteristics in relation to colonization and regulation of host response (3–5). Therefore, identification of the primary structure of capsular polysaccharides produced by members of the genus *Lactobacillus* may provide valuable information on the functional properties of EPS.

*Lactobacillus johnsonii* FI9785 is a poultry-derived isolate that is being investigated as a potential probiotic that may be given to poultry for use as a competitive exclusion agent to control *Clostridium perfringens* (6). *C. perfringens* is a cause of human food poisoning, but some strains are also responsible for necrotic enteritis in poultry, causing problems of animal welfare as well as huge economic losses to the poultry industry worldwide. *L. johnsonii* FI9785 has been shown to adhere well to tissue culture and chick gut explant tissues, out-competing pathogenic bacteria in challenge models. However, the mode of action by which *L. johnsonii* FI9785 achieves this protective effect is unknown.

**FIGURE 1.**
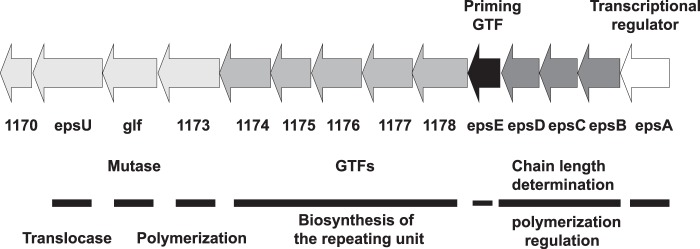
**Molecular organization of the *eps* cluster of *L. johnsonii* FI9785.** The cluster has 14 genes that are predicted to encode a transcriptional regulator (*epsA*), a polymerization and chain length determination protein (*epsB*), a tyrosine-protein kinase (*epsC*), a protein-tyrosine phosphate phosphohydrolase (*epsD*), the priming glycosyltransferase UDP-phosphate galactosephosphotransferase (*epsE*) and five glycosyltransferases (*1178–1174*), an oligosaccharide repeat unit polymerase (*1173*), a mutase (*glf*), an oligosaccharide translocase (*epsU*), and an EPS biosynthesis protein (*1170*) (9).

*L. johnsonii* 142 and *L. johnsonii* NCC533 have also been shown to produce capsular EPS, and deletion of the *eps* cluster in the strain NCC533 resulted in an acapsular phenotype and affected residence time in the murine gut (7, 8). Little is known about the function of the capsular EPS and the mechanism of the biosynthesis for the genus *Lactobacillus*. Previously, the genome of *L. johnsonii* FI9785 was shown to include a 14.9-kb region that harbors 14 putative genes that may be responsible for the EPS biosynthesis in this strain (Fig. 1) (9). The predicted roles of these genes include regulation of sugar biosynthesis, chain length determination, biosynthesis of the repeating unit, polymerization, and export. This cluster has six putative genes encoding glycosyltransferases, which transfer a sugar moiety to the activated acceptor molecule (2, 10). On the basis of homology to conserved domains, the product of the first glycosyltransferase gene, *epsE*, was predicted to initiate the capsular EPS biosynthesis by adding the first sugar to the undecaprenylphosphate, whereas another gene in this cluster, *epsC*, was predicted to encode a tyrosine-protein kinase involved in regulation of capsular EPS biosynthesis (Fig. 1). Changes in the *eps* cluster resulted in alterations in the accumulation level of EPS in derivatives of *L. johnsonii* FI9785; a Δ*epsE* deletion mutant was still able to produce EPS but in lower quantities, whereas an increase in EPS production was observed for a spontaneous *epsC^D88N^* mutant (9). In order to understand the changes in EPS production after these mutations, knowledge of the primary structure of the EPS produced by the wild type and derivative strains is a prerequisite.

In the present study, we identified the structure of two different capsular EPS produced by *L. johnsonii* FI9785. We also investigated strains with mutations in specific genes of the *eps* cluster to examine effects on the structure and biosynthesis of these EPS polymers as well as on the cell surface structure of *L. johnsonii* FI9785. Moreover, we confirmed the localization of specific sugar residues *in situ*. These characterizations may help us to identify the importance of the structure of the capsular EPS to the bacterial cell surface, which may have an impact on colonization and pathogen exclusion by commensal resident gut bacteria.

## EXPERIMENTAL PROCEDURES

### 

#### 

##### Bacterial Strains and Culture Conditions

*L. johnsonii* FI9785 wild type strain and its derivatives, described previously (9) or produced in this study, are listed in Table 1. All strains were grown under static conditions at 37 °C in MRS broth (9) with 2% filter sterilized glucose as the carbon source. To select and maintain plasmids, chloramphenicol (Roche Applied Science) was added at 7.5 μg/ml.

**TABLE 1:** **Bacterial strains used in this study and their EPS content**

Strain	Genotype	Description	EPS content*^a^*	Source
*L. johnsonii* FI9785	Wild type	Wild type strain	832 ± 36	Ref. 9
*L. johnsonii* FI10386	*epsC^D88N^*	one bp change in *epsC* gene	968 ± 34	Ref. 9
*L. johnsonii* FI10844	Δ*epsE*	*epsE* gene deleted	638 ± 41	Ref. 9
*L. johnsonii* FI10773	*epsC^D88N^*::*pepsC*	FI10386 with wild type *epsC* in expression plasmid pFI2560	1082 ± 47	Ref. 9
*L. johnsonii* FI10878	Δ*epsE*::*pepsE*	FI10844 with *epsE* in sense orientation in plasmid pFI2560	920 ± 53	Ref. 9
*L. johnsonii* FI10879	Δ*epsE*::*pepsEA/S*	FI10844 with *epsE* in antisense orientation in plasmid pFI2560	638 ± 64	Ref. 9
*L. johnsonii* FI10754	Δ*eps*_*cluster*	*eps* gene cluster deleted		This study

*^a^* μg/10^9^ cells measured by GC, mean of triplicate samples ± S.D. (9).

##### Deletion of the eps Gene Cluster

The entire *eps* cluster was deleted using a previously described method with some modifications (8). The chloramphenicol resistance gene from plasmid pUK200 (11) was amplified using Phusion polymerase (Finnzymes) with primers CAT_XHOF (5′-AACTCGAGCACCCATTAGTTC-3′) and CATR_SPLICE1170 (5′-AGTACTGTCCTTTACTAACGGGGCAGGT-3′), introducing a XhoI restriction site and a tail for splice overlap extension PCR with sequence from the *FI9785*_*1170* gene (altered nucleotides underlined throughout). The first 390 bp of the *epsA* gene and some upstream sequence was amplified using primers 5epsA_KpnF (5′-AAAGGTA*C*CAAATTAAATAACAAGAG-3′) and epsA_R1 (5′-CGGTAAGTTAACTTTCATATCTCG-3′). The partial *epsA* product was then restricted and ligated into KpnI/XhoI-restricted pG+host9 (12) using Fastlink DNA ligase (Epicenter). The ligation product was transformed into electrocompetent *Escherichia coli* MC1022, and positive colonies were selected with erythromycin (400 μg/ml) and confirmed by colony PCR using GoTaq polymerase (Promega) and primers pGhost1 (5′- AGTCACGACGTTGTAAAACGACG-3′) and pGhostR (5′-TACTACTGACAGCTTCCAAGG-3′). Plasmids were extracted using a plasmid minikit (Qiagen) and sequenced to confirm the partial *epsA* gene insertion. The final construct was named pG+host9epsAp. To amplify the partial *FI9785*_*1170* gene with 280 bp of non-coding region, primers 1170F_SPLICECAT (5′-ACCTGCCCCGTTAGTAAAGGACAGTACT-3′) and 1170_ncR (5′-TATTAAGCTTTCCATTTCCTGC-3′) were used, introducing a tail for splice overlap extension PCR with the chloramphenicol resistance gene product and incorporating a HindIII restriction site, respectively. The products from these two reactions were then used as templates for splice overlap extension PCR together with the primer pair CAT_XHOF and 1170_ncR to produce a 1585-bp product. This was then digested with XhoI and HindIII and subcloned as before into pG+host9epsAp. The deletion plasmid was transformed into *L. johnsonii* FI9785 by electroporation (13), and the method of gene replacement was performed as described by Denou *et al.* (8). The transformants were selected on MRS plates supplemented with chloramphenicol at 30 °C as the permissive temperature for plasmid replication followed by inoculation in MRS broth supplemented with chloramphenicol (7.5 μg/ml) at 42 °C as the non-permissive temperature for five serial passages. The culture was diluted and plated on MRS containing chloramphenicol at 42 °C to obtain single colonies that were replica-plated onto MRS agar with chloramphenicol and MRS with erythromycin to identify Ery^S^, Cm^R^ clones. A positive clone was selected, and the deletion of the *eps* cluster was confirmed by PCR (*L. johnsonii* Δ*eps*_*cluster*).

##### Transmission Electron Microscopy (TEM)

100 μl of 25% glutaraldehyde was added to a 1-ml bacterial suspension in an Eppendorf tube and left to fix for 1.5 h. The suspensions were centrifuged and washed three times in 0.05 m sodium cacodylate buffer. After the final wash, the cell pellets were mixed 1:1 with molten 2% low melting point agarose (Type VII; Sigma), which was solidified by chilling and chopped into small pieces (∼1 mm^3^). The sample pieces were left overnight in 2.5% glutaraldehyde, 0.05 m sodium cacodylate buffer (pH 7.2). The samples were transferred to a Leica EM TP tissue processor (Leica Microsystems UK Ltd., Milton Keynes) where they were washed; postfixed in 1% osmium tetroxide, 0.05 m sodium cacodylate for 2 h; washed; and dehydrated through an ethanol series (30, 50, 70, 90, and 100% × 2) with 1 h between each change. The samples were infiltrated with a 1:1 mix of LR White medium grade resin (London Resin Company Ltd.) to 100% ethanol, followed by a 2:1 and a 3:1 mix and finally 100% resin, with 1 h between each change. This was followed by two more changes into fresh 100% resin, with periods of 8 h between. Six tissue blocks from each sample were placed into gelatin capsules with fresh resin and polymerized overnight at 60 °C. Sections ∼90 nm thick were cut using an ultramicrotome (Ultracut E, Reichert-Jung) collected on film/carbon-coated copper grids, and stained sequentially with uranyl acetate (saturated in 50% ethanol) and Reynold's lead citrate. Sections were examined and imaged in an FEI Tecnai G2 20 Twin transmission electron microscope at 200 kV.

##### Isolation of Capsular Exopolysaccharides

Exopolysaccharides were isolated from 500-ml cultures of bacteria grown for 2 days at 37 °C in MRS broth as described previously (9). In addition to the capsular EPS isolated from the bacterial cell pellets, the capsular EPS that was retained in the supernatant during the centrifugation steps was also harvested and processed separately. These fractions were designated as pellet and supernatant EPS preparations.

##### Atomic Force Microscopy (AFM); Immobilization of Lectins on AFM Tips

Silicon nitride AFM tips (PNP-TR, Nanoworld AG) were functionalized using a four-step procedure (carried out at 21 °C). The first step involved incubation of the tips in a 2% solution of (3-mercaptopropyl)trimethoxysilane (Sigma-Aldrich) in toluene (dried over a 4-Å molecular sieve) for 1 h, followed by washing with toluene and then chloroform. In the second step, the silanized tips were incubated for 1 h in a 0.1% solution of a heterobifunctional linker, MAL-PEG-SCM, 2 kDa (Creative PEGWorks) in chloroform. Unbound linker was washed off with chloroform, and the tips were dried with argon. The third step involved covalent attachment of a lectin from *Pseudomonas aeruginosa* (PA1; Sigma-Aldrich) by incubation of the tips in 1 mg/ml solutions of the lectin in phosphate-buffered saline (PBS) at pH 7.4 for 1 h at 21 °C, followed by a PBS washing step. The fourth step involved incubation of the lectin-functionalized cantilevers in a 10 mg/ml solution of glycine in PBS to “amine”-cap any unreacted succinimide groups, followed by washing in PBS. Lectin-functionalized tips were stored under PBS at 4 °C overnight before use.

##### Immobilization of EPS on Glass Slides

Extracted EPS samples were covalently attached to glass slides using the procedure described above but with a different intermediate linker. The glass was initially functionalized with (3-mercaptopropyl)trimethoxysilane, and then a 2 mm solution of a carbohydrate-binding heterobifunctional linker γ-maleimidophenylbutyric acid hydrazide hydrochloride in methanol was incubated on the slide for 1 h at 21 °C, followed by a methanol rinsing step. Next, solutions of the extracted EPS samples (0.1% in PBS) were incubated on the slides for 1 h at 21 °C and then rinsed with PBS. Finally, slides were incubated in 10 mg/ml solutions of glucose in PBS to sugar-cap any remaining unreacted hydrazide groups. Force mapping measurements on the EPS-coated slides were carried out as below.

##### Force Mapping Measurements

Bacterial cells were electrostatically attached to glass slides to enable force mapping to be carried out in aqueous buffer. Freshly washed glass slides were incubated in a 0.01% solution of poly-l-lysine (Sigma-Aldrich) for 5 min at 20 °C. Treated slides were drained and dried for 1 h at 60 °C and then allowed to cool to room temperature. Bacterial cell suspensions (∼10^8^ cells/ml) in distilled water were incubated on the treated slides for 1 h. The slides were rinsed with distilled water to remove any non-adherent cells, and excess liquid was removed before insertion into the liquid cell of the atomic force microscope, where they were immersed in PBS. All binding measurements on cell surfaces were carried out under PBS using a MFP-3D BIO atomic force microscope (Asylum Research Inc.). The experimental data were captured in “force-volume” mode (at a rate of 2 μm/s in the *z* direction and at a scan rate of 1 Hz and a pixel density of 32 × 32). In this mode, the instrument ramps the *z* piezo element of the scanner by a predetermined amount at each sample point over a selected scan area and records the subsequent deflection of the cantilever as it is pushed into (maximum load force, 300 pN) and then retracted away from the sample surface. This produces a matrix of 1024 force *versus* distance curves relating to the image coordinates. The spring constant, *k*, of the cantilevers was determined by fitting the thermal noise spectra (14), yielding typical values in the range 0.01–0.04 newtons/m. Adhesion in force spectra was quantified using a bespoke Excel macro (15), which fits a straight line to the base line of the retract portion of the force-distance data, and wormlike chain fitting of the adhesion peaks was performed using a routine in the instrument's software.

##### Production of Anti-wild Type Antibodies

*L. johnsonii* FI9785 was grown in MRS, and the cells were inactivated with 1% formalin and incubated for 30 min at room temperature. Inactivated cells were dialyzed against PBS. Polyclonal anti-wild type antibodies were raised in rabbits by BioGenes (Germany) to a titer of >1:200,000. The specificity of the antibody was tested by ELISA (16).

##### Immunodetection of Bacterial Surface Changes by Flow Cytometry

Wild type and derivative strains were grown to stationary phase, washed twice in PBS, and resuspended in PBS to an optical density (*A*_600_) of 1.0. Cells were transferred (100 μl/well) onto a normal binding microtiter plate (Greiner Bio-One); BSA (1 mg/ml in PBS) was included as a negative control. 25 μl of diluted antibody (1:200 in PBS) was added per well and incubated at room temperature for 30 min. 175 μl of PBS was added to each well, the plate was centrifuged at 4000 × *g* for 15 min, and the pellet was resuspended in 100 μl of fluorescein-conjugated goat anti-rabbit IgG (Sigma-Aldrich) (1:750 in PBS) solution. The antibody-bacteria complexes were then incubated at room temperature for 15 min. PBS (200 μl) was added to each well, and the antibody responses to the strains were measured as the median fluorescence from the green fluorescein, detected via PMT sensors in channel FL1 (530/30) at 568–583 nm in a FC500 cytometer (Beckman Coulter). A total of 20,000 events/sample were acquired at a low rate. Flow cytometry data were analyzed using FlowJo (TreeStar).

##### NMR Spectroscopy Analysis

NMR samples were prepared by adding 600 μl of D_2_O to ∼1 mg of each lyophilized polysaccharide, followed by vigorous mixing and centrifugation. Supernatants (550 μl) were transferred to 5-mm NMR tubes. Spectra were measured at 600 MHz (^1^H) and 150 MHz (^13^C) using a Bruker Avance 600 NMR spectrometer equipped with a TCI cryoprobe. Sample temperature was set at 300 K for an initial ^1^H NMR screening of all samples and at 338 K for subsequent two-dimensional and ^13^C NMR studies of the wild type, *epsC^D88N^*, and Δ*epsE* samples. The 90° pulses were 9.1 μs (^1^H) and 10 μs (^13^C), and spectra were acquired with presaturation of the residual HDO signal using standard Bruker methods and parameters (name of the pulse sequence is shown in italic type, followed by the number of scans for each experiment (NS)): ^1^H (*noesygppr1d*, NS = 64); ^13^C (*zgpg30*, NS = 20,000); COSY (*cosygpmfqfpr*, NS = 32); TOCSY (*mlevphpr.2*, NS = 32, mixing time = 100 ms); ROESY (*roesyphpr*, NS = 24, mixing time = 400 ms); HSQC (*hsqcetgpprsisp2.2,* NS = 64); HMBC (*hmbcgplpndprqf*, NS = 64); HSQC-TOCSY (*hsqcdietgpsisp.2*, NS = 128, mixing time = 150 ms).

Homonuclear experiments were run with spectral widths of 12 ppm in both dimensions (or 3.5 ppm for higher resolution in TOCSY and ROESY); heteronuclear experiments were run with spectral widths of 12 ppm (^1^H) × 166 ppm (^13^C HSQC, HSQC-TOCSY) or 250 ppm (^13^C HMBC) acquired into 2048 (TD) × 256 matrices and Fourier transformed with zero filling into 2048 × 1024 matrices. Spectra were referenced to the methyl signal of DSS (δ^1^H = 0 ppm, δ^13^C = 0 ppm) via the methyl signal of ethanol (present as an impurity in all samples) at δ^1^H = 1.18 ppm and δ^13^C = 19.59 ppm with respect to DSS. Note that on this scale, the chemical shifts of acetone are (δ^1^H = 2.208 ppm, δ^13^C = 32.69 ppm) and will be different from the values used by many authors in carbohydrate NMR (17).

##### Solid State NMR Spectroscopy

EPS samples were hydrated and loaded in 4-mm MAS NMR rotors. Solid-state NMR experiments were carried out on a Varian 400-MHz VNMRS direct drive spectrometer with a 4-mm T3 MAS NMR probe (Varian Inc.). Temperature was regulated using balanced heated/vortex tube-cooled gas flow (18). All ^31^P spectra were referenced externally to 10% H_3_PO_4_ at 0 ppm. Spectra were acquired at 2 °C under 12-kHz MAS following 104-kHz direct excitation ^31^P pulse (π/2 = 2.4 μs) without proton decoupling, and 8192 transients were averaged in acquisition. The interpulse delay was set to 5 s, but in some experiments, it was extended to 30 s to ensure uniform excitation, including putative long *T*_1_ species. Longitudinal relaxation times were determined for assigned resonances using inversion recovery with 104-kHz pulses and relaxation delays of 0.001, 0.01, 0.1, 1, 3, and 5 s, and the repeat time was set at 15 s. Spectra were processed and analyzed using ACD/Labs (Advanced Chemistry Development Inc.). Individual resonances were approximated by simultaneous fitting to Gauss-Lorentzian line shapes.

## RESULTS

### 

#### 

##### Structural Analysis of EPS by NMR Spectroscopy

To investigate the role of specific genes of the *eps* cluster in capsular EPS biosynthesis and production level, we compared the structure of capsular EPS isolated from the wild type, the Δ*epsE* deletion mutant, and the *epsC* single base pair mutant and their complemented strains as well as the Δ*eps*_*cluster*, where the entire 14.6-kb gene cluster was removed. None of the changes in the *eps* cluster affected the growth rate of *L. johnsonii* strains (data not shown). Two types of EPS extracts were prepared, cell surface-associated (“pellet”) and EPS extracted from the supernatant (“supernatant”). EPS was harvested from all strains; EPS extractions from the Δ*eps*_*cluster* strain gave a much lower yield of the final freeze-dried product, but the sample was treated in the same way and subjected to NMR analysis with the other samples.

**FIGURE 2.**
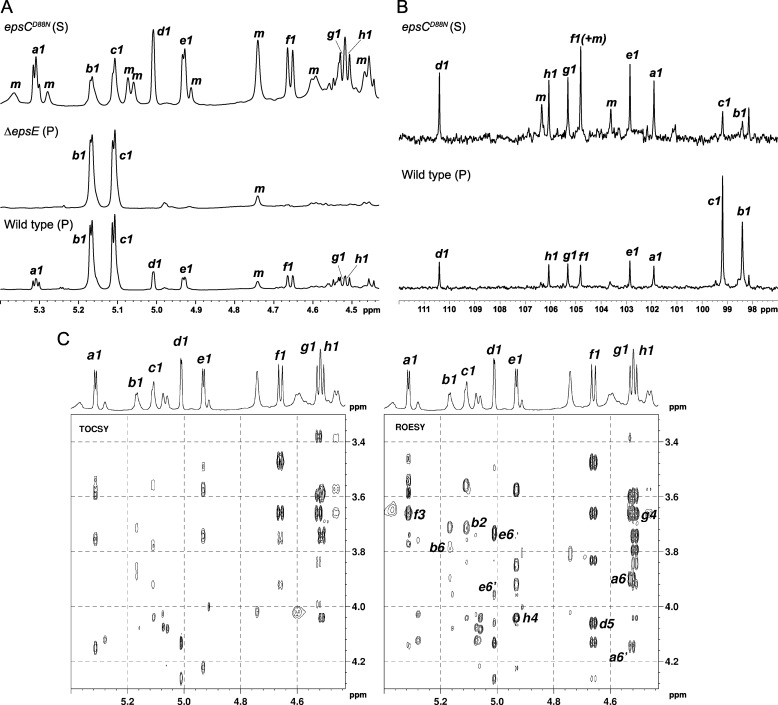
**NMR analysis shows two novel exopolysaccharides.** *A*, 600-MHz ^1^H NMR spectra (anomeric region, 338 K, D_2_O) of exopolysaccharides produced by *L. johnsonii* FI9785 and two mutant strains. Sugar units *b* and *c* are from EPS-1, and units *a* and *d–h* are from EPS-2. Peaks labeled *m* are from the growth medium, those labeled *S* are from the supernatant fraction, and those labeled *P* are from the pellet fraction. *B*, 150-MHz ^13^C NMR spectra (anomeric region, 338 K, D_2_O) of exopolysaccharides produced by *L. johnsonii* FI9785 and a mutant strain. Sugar units *b* and *c* are from EPS-1, and units *a* and *d–h* are from EPS-2. Peaks labeled *m* are from the growth medium. *C*, 600-MHz two-dimensional NMR spectra (338 K, D_2_O) of exopolysaccharides from *L. johnsonii epsC^D88N^* (*S*). *Left*, TOCSY spectrum showing coupling networks associated with each anomeric signal; *right*, ROESY spectrum. *Labels* indicate hydrogens brought into proximity across glycosidic linkages (*a*1*–f*3, *c*1*–b*2, etc.).

An initial screening of all pellet and supernatant EPS samples by ^1^H NMR at 300 K showed that two anomeric signals at 5.17 and 5.11 ppm were a major feature of all cell surface-associated (pellet) EPS preparations. These signals were also present in the supernatant series, although in most cases, they were no longer the major ones in the anomeric region. The polysaccharide sugar rings were partially acetylated because a cluster of at least six methyl singlet signals was observed between 1.98 and 2.08 ppm plus, in some samples, an isolated singlet at 2.16 ppm. Representative samples were selected for detailed NMR studies, and for these, the temperature was increased to 338 K as a significant sharpening of ^1^H signals was obtained (Fig. 2*A*) (*e.g.* the apparent singlets at 5.17 (labeled *b*1) and 5.11 ppm (*c*1) were revealed as doublets); also, the residual HDO signal (4.41 ppm) did not interfere with any other peaks at this temperature.

The ^1^H and ^13^C NMR spectra of the representative samples (anomeric regions shown in Fig. 2, *A* and *B*) also confirmed that *L. johnsonii* FI9785 produced a mixture of two exopolysaccharides; in particular, the pattern of intensities found in the different samples suggested that the two signals labeled *b*1 and *c*1 belonged to one polysaccharide (EPS-1), whereas the six signals labeled *a*1 and *d*1*–h*1 belonged to a second one (EPS-2). The signals were labeled *a–h* in descending order of ^1^H chemical shift, as shown in Fig. 2*A*; the correlation between the directly linked ^1^H and ^13^C atoms was established using the HSQC spectrum and was used to label the ^13^C anomeric signals (Fig. 2*B*). Integration of the ^1^H and ^13^C anomeric regions showed that the EPS-1 repeating unit was made up of two sugar units, present in equal amounts (the ^13^C signal of *b*1 is slightly broader than that of *c*1, accounting for the difference in signal heights); the EPS-2 repeating unit contained six different sugar units. Signals labeled *m* were found in control samples prepared from medium that had not been inoculated with bacteria and will not be discussed further. The structures of EPS-1 and EPS-2 were determined using a combination of two-dimensional NMR methods: COSY, TOCSY, HSQC, and HSQC-TOCSY, to assign the ^1^H and ^13^C chemical shifts within each sugar ring and ROESY and HMBC to determine the sequence of the sugars and their linkage positions. Results of the ROESY and HMBC experiments are summarized in Table 2, and the chemical shifts of the two polysaccharides are reported in Table 3 (EPS-1) and Table 4 (EPS-2).

**TABLE 2:** **Connectivities between the anomeric ^1^H signal of each ring and other resonances revealed by ROESY and HMBC experiments** Boldface numbers indicate δ^1^H (or δ^13^C) of atoms involved in glycosidic linkages.

Anomeric		ROE, δ^1^H (label)	HMBC, δ^13^C (label)
Label	δ^1^H		
	*ppm*	*ppm*	*ppm*
*a*1	**5.31**	3.46 (*f*2), **3.66** (*f*3)	**85.67** (*f*3)
*b*1	**5.17**	**3.78** (*b*6), 5.11 (*c*1)	**68.66** (*b*6)
*c*1	**5.11**	3.56 (*c*2), **3.71** (*b*2), 5.17 (*b*1)	**78.62** (*b*2)
*d*1	**5.01**	**3.73** (*e*6), **3.96** (*e*6′)	**69.26** (*e*6)
*e*1	**4.93**	3.58 (*e*2), 3.85/3.92 (*h*6/6′), **4.04** (*h*4)	**80.38** (*h*4)
*f*1	**4.66**	3.48 (*f*5), 3.66 (*f*3), 3.83 (*d*6), **4.06** (*d*5), 4.13 (*d*4)	**80.55** (*d*5)
*g*1	**4.53**	3.60 (*g*5), 3.66 (*g*3), **3.90** (*a*6), **4.14** (*a*6′)	**71.22** (*a*6)
*h1*	**4.51**	3.60 (*h*2), **3.66** (*g*4), 3.74 (*h*3), 3.79 (*h*5), 3.84/3.92 (*h*6/6′)	**81.80** (*g*4)

**TABLE 3:** **^1^H and ^13^C chemical shifts of EPS-1 repeating unit**

Label	Unit		Chemical shift					
			1	2	3	4	5	6
			*ppm*					
*b*	(1,2,6)αGlc*p*→6	H	5.17	3.71	3.86	3.62	3.89	3.78, 4.03
		C	98.42	78.62	74.55	72.38	73.09	68.66
*c*	*t*-αGlc*p*→2	H	5.11	3.56	3.77	3.45	3.92	3.78, 3.86
		C	99.21	74.22	75.83	72.38	74.79	63.42
	1-Phosphoglycerol*^a^*	H	3.90, 3.97	3.91	3.62, 3.69			
		C	69.11	73.54	65.11			

*^a^* Partial substituent on unit *c*. Substituted unit *c*: H5/C5 = 4.02/73.77 ppm; H6/C6 = 4.11/67.0 ppm

**TABLE 4:** **^1^H and ^13^C chemical shifts of EPS-2 repeating unit** Rows follow the same order as sugars in EPS-2 repeating unit with *g* linked to *a*.

Label	Unit		Chemical shift					
			1	2	3	4	5	6
			*ppm*					
*a*	(1,6)αGlc*p*→3	H	5.31	3.59	3.75	3.54	4.16	3.89, 4.14
		C	101.95	74.43	75.74	72.18	73.80	71.22
*f*	(1,3)βGlc*p*→5	H	4.66	3.46	3.66	3.66	3.48	3.76, 3.93
		C	104.83	74.78	85.67	72.78	78.27	63.55
*d*	(1,5)βGal*f*→6	H	5.01	4.13	4.27	4.12	4.05	3.83
		C	110.42	83.62	79.03	84.42	80.55	63.98
*e*	(1,6)αGlc*p*→4	H	4.93	3.58	3.74	3.49	4.22	3.73, 3.96
		C	102.86	74.50	75.53	72.38	73.80	69.26
*h*	(1,4)βGal*p*→4	H	4.51	3.59	3.74	4.04	3.79	3.84, 3.92
		C	106.09	73.80	75.04	80.38	78.11	63.07
*g*	(1,4)βGlc*p*→6	H	4.53	3.39	3.66	3.66	3.60	3.84, 4.00
		C	105.33	75.53	77.16	81.80	77.54	63.04

##### EPS-1

**FIGURE 3.**
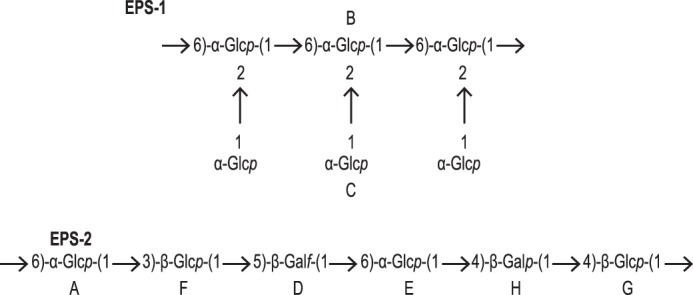
**Structure of exopolysaccharides EPS-1 and EPS-2.** The sugar rings in EPS-1 and EPS-2 are labeled *A–H*, and these *letters* correspond with the (*lowercase*) labeling of the NMR signals in Fig. 2.

The structure of EPS-1 was determined mainly from experiments on the wild type (WT-bacterial pellet) sample. Rings *b* and *c* were found to be both α-Glc*p*; *b*1 and *c*1 had ^3^J_12_ = 3.5 Hz, consistent with α configuration. In both rings, H1 was linked to H5 through all intermediate protons in the TOCSY experiment, and the shapes of the cross-peaks indicated substantial couplings throughout, as expected for Glc*p*. The HSQC-TOCSY experiment linked H1 for each ring to all carbons of the same ring, including C6. In particular, *b*1 and *c*1 were linked to C6 signals at 68.66 and 63.42 ppm, respectively. Chemical shifts of EPS-1 are reported in Table 3. The connectivities (Table 2) showed that EPS-1 consists of a chain of α-(1,6)-linked Glc*p* residues (ring *b*), all of which are additionally substituted at position 2 with a single α-Glc*p* (ring *c*), as shown in Fig. 3. The chemical shifts of rings *b* and *c* are close to those reported for (1,2,6)α-Glc*p* and *t*-α-Glc*p* in a dextran isolated from *Leuconostoc citreum* E497 (19); however, EPS-1 contained none of the unbranched (1,6)α-Glc*p* residues that were the major constituents of the *L. citreum* E497 dextran backbone.

##### EPS-2

The structure was determined mainly from the *epsC^D88N^* (supernatant) sample. Four of the six sugar units were readily identified as Glc*p* on the basis of the TOCSY spectrum (Fig. 2*C*), in which all four had coupling networks extending from H1 to H5 and, more weakly, to H6 (in some rings, only one H6 was visible, the other being obscured by overlap). The two anomeric signals *a*1 and *e*1 (both ^3^J_12_ = 3.5 Hz) were associated with α-Glc*p*, whereas *f*1 and *g*1 (both ^3^J_12_ = 7.9 Hz) belonged to β-Glc*p* units. All ^13^C chemical shifts within each Glc ring could be determined by HSQC-TOCSY, including C6. The downfield shifts of two C6 resonances (*a*6 = 71.22 ppm and *e*6 = 69.26 ppm, relative to *f*6 = 63.55 ppm and *g*6 = 63.04 ppm) indicated that the two α-Glc*p* units were 6-linked. Similarly, the downfield shifts of C3 in ring *f* (*f*3 = 85.67 ppm) and C4 in ring *g* (*g*4 = 81.80 ppm) indicated that the β-Glc*p* units, *f* and *g*, were 3- and 4-linked, respectively. A fifth sugar unit with anomeric signal *h*1 (^3^J_12_ = 7.3 Hz) was identified as β-Gal*p* because the TOCSY coupling network from *h*1 terminated with a narrow cross-peak (^3^J_34_ small and ^3^J_45_ = 0 Hz) at *h*4 = 4.04 ppm. The remaining chemical shifts (*h*5, *h*6/6′) were determined from the ROESY spectrum. The ^13^C shift of *h*4 = 80.38 ppm pointed to a 4-linked β-Gal*p* unit. Chemical shifts of the sixth sugar unit (*d*1 = 4.93 ppm, ^3^J_12_ = 2 Hz) could be assigned from the combined two-dimensional NMR experiments; the presence of six ^13^C signals in the HSQC-TOCSY spectrum indicated that ring *d* was a hexose. However, the anomeric carbon (*d*1 = 110.42 ppm) as well as the *d*2–*d*4 ^1^H and ^13^C chemical shifts were found considerably downfield of the typical values expected for pyranose rings (excluding linkage positions), suggesting that *d* was probably a furanose residue. The EPS produced by *L. johnsonii* 142 was reported to contain a (1,5)-β-Gal*f* (galactofuranose) residue (7), and it had NMR parameters similar to those of *d* in Table 4. We also found from the ROESY and HMBC experiments that *d* was 5-linked (Table 2), so we conclude that *d* in EPS-2 is a (1,5)-β-Gal*f* unit. The proposed linkage positions (*x*) in all rings were confirmed by the detection in ROESY and HMBC spectra of H_1_^′^C_1_′OC*_x_*H*_x_* and H_1_′C_1_′OC*_x_* interresidue cross-peaks that were not present in the TOCSY or HSQC-TOCSY spectra (see Fig. 2*C* for TOCSY and ROESY spectra of *epsC^D88N^*). These additional connectivities also allowed the sequence of sugar residues in the hexasaccharide repeating unit of EPS-2 to be determined as shown in Fig. 3 and Table 4.

The composition of the EPS mixtures produced by the wild type, the *epsC^D88N^* and Δ*epsE* mutants, and their complemented strains could be readily assessed from the anomeric region of the ^1^H NMR spectra following the unequivocal assignment of signals to EPS-1 and EPS-2. The wild type, *epsC^D88N^*, and its complemented strain produced both EPS-1 and EPS-2, whereas Δ*epsE* and its derivative strain containing the wild type gene in the antisense orientation produced only the dextran, EPS-1. However, the ability to produce EPS-2 as well as EPS-1 was restored in the Δ*epsE* strain complemented with the wild type *epsE* gene. Importantly, the Δ*eps*_*cluster* mutant was unable to produce either EPS-1 or EPS-2 (data not shown).

##### Substituent Groups

We did not attempt to determine the locations of all of the acetyl groups; the major signals arise from non-acetylated sugar rings, and the reported chemical shifts in Tables 3 and 4 correspond to these. However, we found that the acetyl group that gave rise to the isolated ^1^H signal at 2.16 ppm was lost upon extended storage of the *epsC^D88N^* (supernatant) sample. It showed that this acetyl group was present in EPS-2 because its loss was accompanied by minor changes elsewhere in the EPS-2 spectrum (*e.g. a*1, which appears as two unequal intensity doublets in Fig. 2*A*, becomes a simple doublet after loss of the acetyl group). The Δ*epsE* mutant that lacked EPS-2 still had the cluster of signals at 1.98–2.08 ppm (not the 2.16 ppm signal), and therefore the acetyl groups that give rise to that cluster must be associated with EPS-1. Integration of the ^1^H spectrum of Δ*epsE* showed that the total level of *O*-acetyl group substitution in EPS-1 amounted to about 0.3 of one -OH group, with substituents distributed unequally across the seven available -OH groups.

**FIGURE 4.**
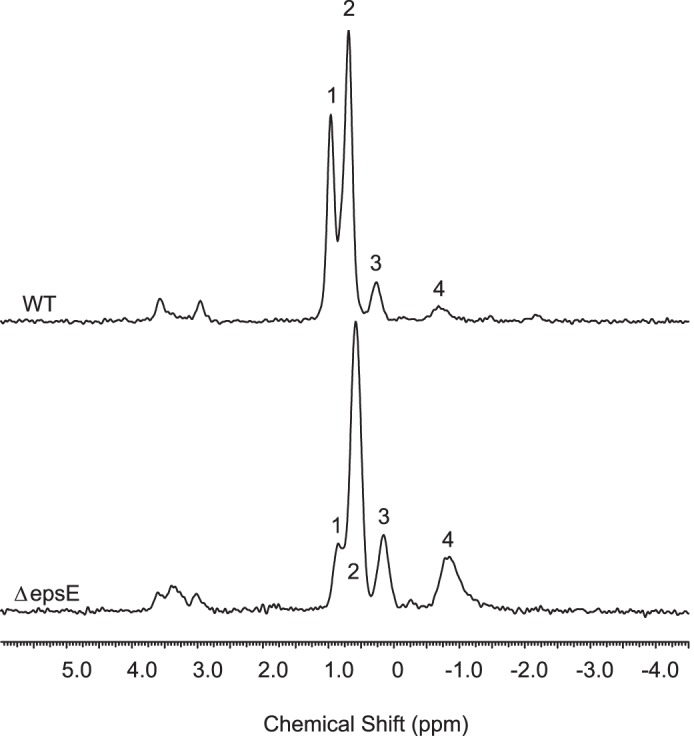
**Solid state ^31^P MAS NMR spectra of EPS from wild type *L. johnsonii* and from the Δ*epsE* mutant.** Peak numbering corresponds to listing in Table 5.

WT and Δ*epsE* mutant samples were also investigated by high resolution ^31^P MAS solid state NMR; WT (∼75% EPS-1, 25% EPS-2) and Δ*epsE* (∼100% EPS-1) showed multiple peaks (Fig. 4), most of which were common to both spectra (Table 5). Given that ^1^H and ^13^C spectra showed the presence of impurities, we cannot exclude the possibility that impurities are also responsible for some of the ^31^P signals. However, ^13^C and HSQC spectra of the Δ*epsE* mutant revealed the presence of the 1-phosphoglycerol substituent (20) at a level of about 0.2 of one -OH group in EPS-1. Characteristic ^13^C signals for the 1-phosphoglycerol group were C1 69.1 ppm (CH_2_, d, ^2^J_PC_ = 5.7 Hz); C2 73.5 ppm (CH, d, ^3^J_PC_ = 7.4 Hz); C3 65.1 ppm (CH_2_, s) with associated ^1^H signals (Table 4) in excellent agreement with those reported for the 1-phosphoglycerol unit reported in the EPS produced by *Lactobacillus paracasei* 34-1; also, the major ^31^P signal (0.6 ppm) in Δ*epsE* is close to that reported (0.88 ppm) in *L. paracasei* 34-1 EPS, which is stated to be typical of a phosphodiester (20). ^13^C signals from the 1-phosphoglycerol group were also present in the WT (predominantly EPS-1) spectrum but were not found in the spectrum of another mutant (data not shown), which produced essentially only EPS-2. Therefore, the 1-phosphoglycerol substituent is associated only with EPS-1; the low level of substitution makes a full assignment of the substituted sugar units difficult, but plausible assignments of minor peaks in the ^13^C and two-dimensional spectra of Δ*epsE* suggest that the substituent is located on the *t*-αGlc side chain of EPS-1. The TOCSY spectrum of Δ*epsE* reveals two signals at 4.11 and 4.02 ppm linked to an anomeric signal at 5.10 ppm; the corresponding ^13^C signals from the HSQC/DEPT spectra are at 67.0 ppm (CH_2_, broad s) and 73.77 ppm (CH, d, ^3^J_PC_ = 7.4 Hz) and were assigned as C6 and C5, respectively, of unit *c* carrying a substituent. These signals are not present in the main unsubstituted *t*-αGlc unit *c* with the anomeric signal at 5.11 ppm (Table 3). The minor peaks are consistent with the location of the 1-phosphoglycerol group at C6 of the *t*-αGlc, *c*, producing expected (20) downfield shifts of H6/C6 (4.11/67.0 ppm), an upfield shift of the neighboring C5 (73.77 ppm), and downfield shift of H5 (4.02 ppm). These chemical shifts may be compared with the corresponding values for the unsubstituted unit *c* given in Table 3.

**TABLE 5:** **Solid state ^31^P MAS NMR spectroscopic parameters of hydrated wild type and Δ*epsE* mutant EPS from *L. johnsonii*** All chemical shifts in ppm and integrals were obtained from simultaneous Gauss-Lorentzian fitting of the entire spectra using ADC-Labs. Chemical shifts were externally referenced to 0 ppm for H_3_PO_4_. Longitudinal relaxation times (s) were obtained by inversion recovery. ND, not determined.

WT (EPS-1 and EPS-2)					Δ*epsE* (EPS-1)				
CS_iso_	Integral	Fraction	Peak	*T*_1_	CS_iso_	Integral	Fraction	Peak	*T*_1_
*ppm*				*s*	*ppm*				*s*
−0.68	8	2	4	ND	−0.85	47	11	4	1.45
0.26	9	3	3	1.36	0.16	27	6	3	0.92
0.69	100	33	2	1.02	0.58	100	25	2	1.28
0.96	68	22	1	0.67	0.85	23	5	1	0.87
2.96	6	2			3.01	4	1		
					3.27	5	1		
3.46	3	1			3.34	7	1		
3.57	6	2			3.60	5	1		

##### Transmission Electron Microscopy

**FIGURE 5.**
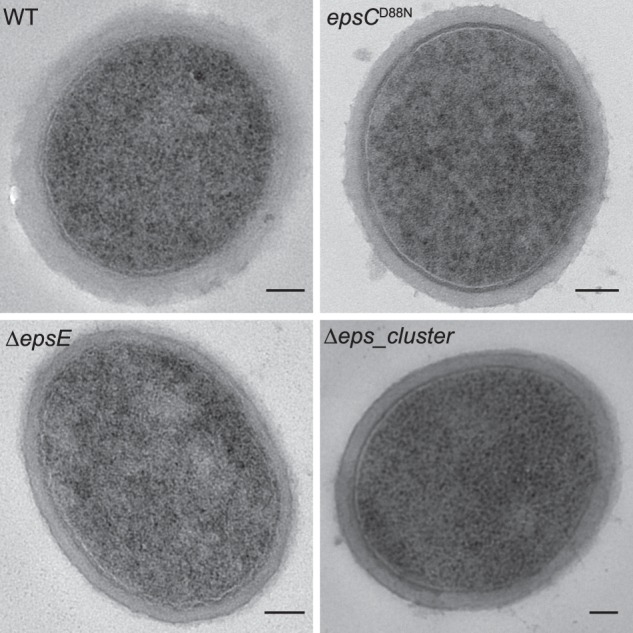
**Accumulation of exopolysaccharides on the cell surface of *L. johnsonii* FI9785 and derivative strains.** TEM analysis of *L. johnsonii* FI9785 and mutant strains in MRS medium showing the variation in the EPS layer. *Bar*, 100 nm.

TEM showed the accumulation of the EPS to the cell surface, where they formed a capsule as an outer cell surface layer in *L. johnsonii* FI9785 (Fig. 5). An EPS layer still accumulated at the cell surface of the Δ*epsE* mutant, consisting solely of EPS-1, whereas the EPS layer was absent from the Δ*eps*_*cluster* mutant (Fig. 5). We analyzed all strains using TEM, but the observed differences in the thickness of the EPS layer did not match the yields of EPS measured in previous work, suggesting that the preparation procedure resulted in the loss of some EPS from the cell surface (Fig. 5) into the culture medium. Washing with buffers that have no EPS cross-linking potential has been reported to remove capsular EPS (21); in particular, the *epsC^D88N^* mutant shown previously to have an increased accumulation of EPS (9) appeared to have a similar or slightly reduced capsule thickness compared with the wild type strain, and this may have implications for the nature of the interactions of the EPS within the capsule and with the cell wall.

##### Antibody Responses Measured by Flow Cytometry

**FIGURE 6.**
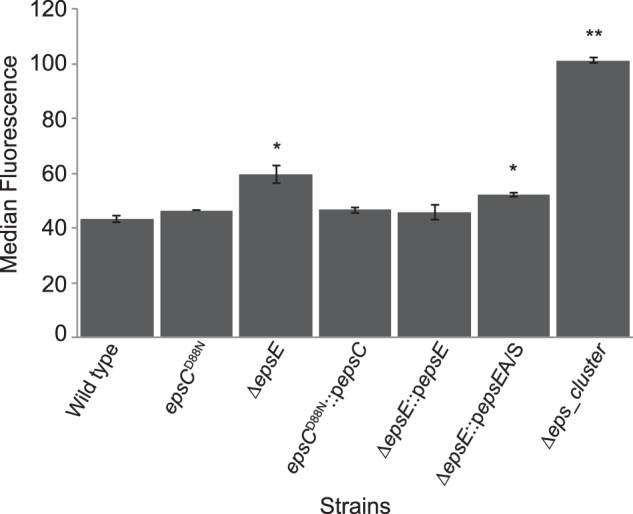
**Anti-wild type antibody responses to the wild type and derivative strains measured by flow cytometry.** Results are the mean of duplicate experiments ± S.D. (*error bars*) Significant differences were determined by an independent *t* test compared with the wild type. *, *p* < 0.05; **, *p* < 0.005.

Flow cytometry has recently become an important tool to detect the antibody responses against live bacteria (22). To investigate the cell surface changes after *eps* mutations, responses to an antibody raised against the whole cells of the wild type FI9785 were detected by using flow cytometry. The median value of the fluorescent signal showed the specific binding of the antibody to each strain. The non-EPS producing strain, the Δ*eps*_*cluster* mutant, showed a significantly higher response to this polyclonal antibody compared with the wild type and the other mutants (Fig. 6). The increase of the antibody response in this deletion strain was around 3 times higher than the antibody response to wild type cells, suggesting the exposure of the cell surface epitopes after loss of the EPS layer. Similarly, the antibody response to the Δ*epsE* mutant was higher than that to the wild type and the other strains except the Δ*eps*_*cluster* mutant. An increased antibody response was also seen in the Δ*epsE*::*pepsEA/S* strain, although to a lesser extent than the Δ*epsE* mutant, whereas the Δ*epsE* strain complemented with the wild type gene showed a similar antibody response to the wild type (Fig. 6). This indicates that although the Δ*epsE* mutant retains an EPS layer, the inability to produce EPS-2 as a capsular material at the cell surface may have resulted in an increased availability of the cell surface epitopes for antibody binding. Despite the increased levels of EPS production in the *epsC^D88N^* mutant and its complemented derivative, the levels of antibody response were similar to the wild type, suggesting that EPS-2 is not highly immunogenic.

##### Atomic Force Microscopy

**FIGURE 7.**
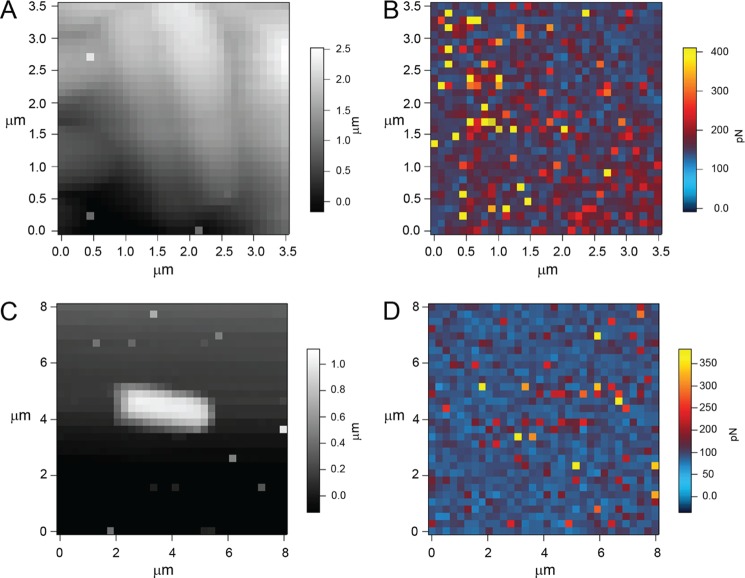
**Force-volume images obtained with a PA1-functionalized AFM tip.** Shown are *L. johnsonii* (wild type) topography (*A*) and adhesion (*B*) as well as *L. johnsonii* (Δ*epsE* mutant) topography (*C*) and adhesion (*D*).

**FIGURE 8.**
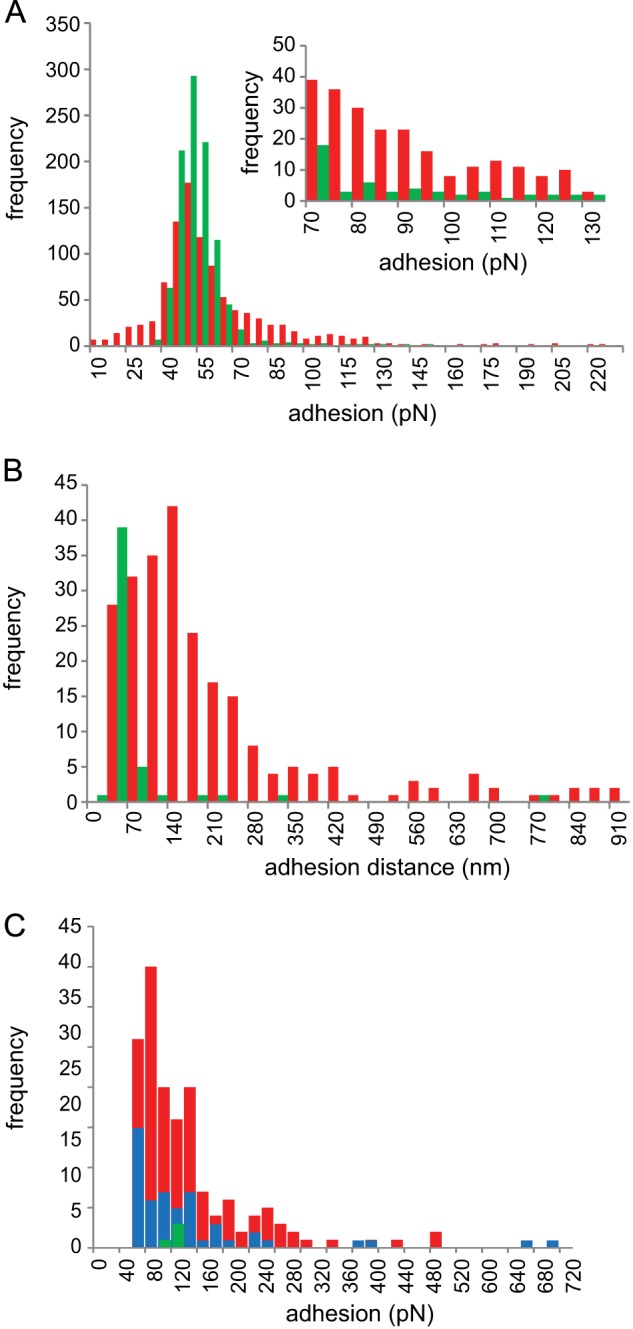
**Adhesion data from force-volume data.** *A*, distribution of rupture force magnitudes using data from Fig. 7 depicted as histograms. *Inset*, expanded view of data >70 pN. *B*, distribution of rupture distances. *C*, distribution of rupture forces obtained from a PA1-functionalized AFM tip probing EPS extracts covalently attached to a glass slide. *Red*, wild type; *green*, Δ*epsE* mutant; *blue*, wild type in galactose solution.

Probing the cell surfaces of two of the *L. johnsonii* strains with a d-galactose-specific lectin (PA1)-functionalized AFM tip allowed an *in situ* discrimination of the different EPS produced, given that EPS-2 has galactose residues that are absent in EPS-1 (Fig. 3). Fig. 7 shows comparative force-volume images of the wild type and Δ*epsE* mutant strains, allowing the topography of the cells to be compared with the adhesive interactions detected. The *left-hand panels* depict topography, and the *right-hand panels* depict the levels of adhesion encountered by the PA-1-functionalized AFM tip at each imaging point. A close-packed cluster of wild type cells (Fig. 7*A*) can be seen, and a single Δ*epsE* mutant cell is visualized (Fig. 7*C*). The adhesion maps reveal that a larger number of the pixels displayed adhesion above the base-line level (∼50 pN) for the wild type sample (Fig. 7*B*) than the Δ*epsE* mutant sample (Fig. 7*D*). Analysis of the adhesion data captured on the two samples allowed a quantitative comparison to be made. The modal value for both samples occurs between 50 and 55 pN (Fig. 8*A*). Although the base-line level of adhesion appears similar for both samples, the wild type data set has a greater proportion of adhesion events in the higher value categories than the Δ*epsE* data set (*inset*), indicating a higher degree of specific interactions.

**FIGURE 9.**
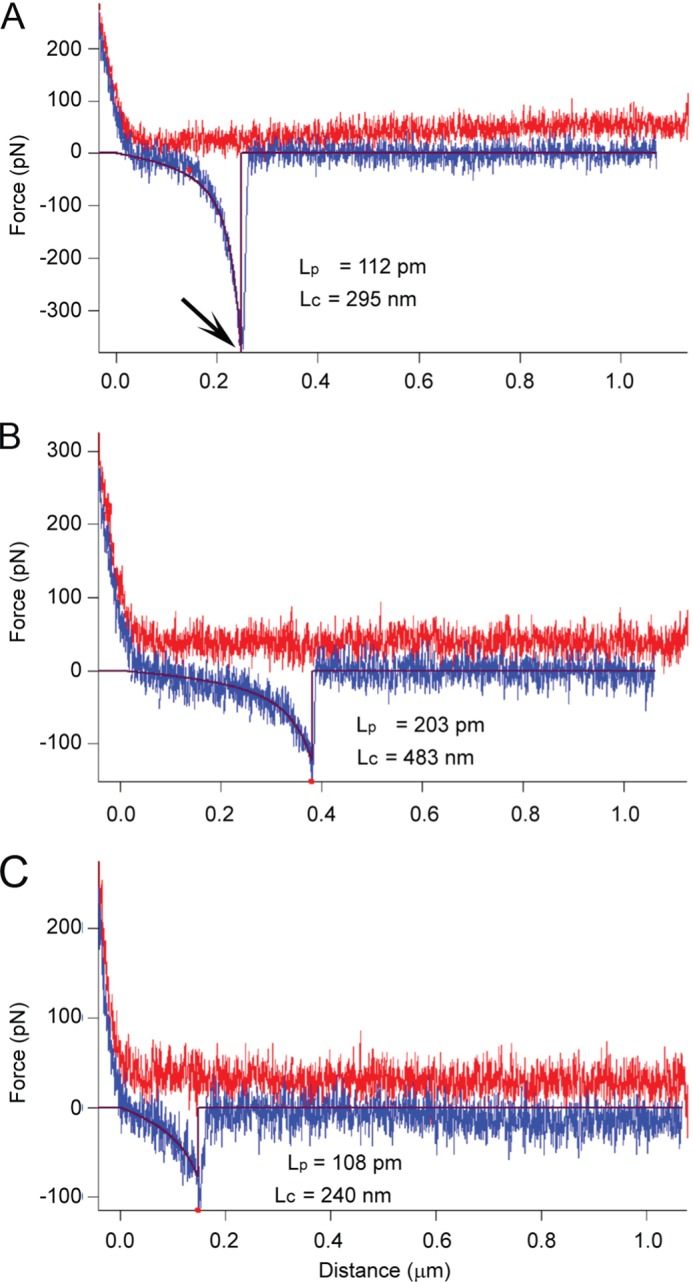
***In situ* characterization of the physical properties of EPS-2.** Example force spectra (*A–C*) from the *L. johnsonii* wild type were fitted to a wormlike chain model (*brown line*). *L*_c_, derived contour length; *L*_p_, derived persistence length. *Arrow*, the rupture point between the lectin on the AFM tip and the extracellular polysaccharide. *Red line*, approach; *blue line*, retract.

The lower base-line adhesion values surrounding the mode in both sets may well be due to nonspecific adhesion between the AFM tip and the cell surfaces. This can arise from several sources; one is electrostatic interaction between the tip and cell, although in the current experiment, this should be minimal due to the screening action of the buffer solution used. Another possible source can be penetration of the AFM tip apex into the bacterial cell wall during the approach phase of the measurement. This causes capillary adhesion as the tip is pulled away from the cell surface. In order to minimize this, the maximum loading force was kept to a moderately low value (300 pN), but some penetration or deformation of the cell surface is inevitable when one considers the sharpness of AFM tips (typical radius of curvature, 5–30 nm), although cells have been shown to tolerate such puncturing (23). Both of these nonspecific sources of adhesion tend to occur at (or relatively close to) the tip-sample detachment point (defined as 0 nm in the force-distance curves), whereas specific adhesion between the lectin on the AFM tip and the EPS will occur at distances well beyond the tip-sample detachment point, allowing discrimination of the origins of adhesive peaks in the force spectra. The reason for the shift in position of specific adhesion is due to two factors; the probe molecule (PA1 lectin) is tethered to the AFM tip via a flexible PEG linker, which is ∼12 nm in length, and the EPS targeted will extend under the load exerted by the retracting AFM tip-cantilever assembly before the ligand and receptor are torn from each other (*i.e.* the rupture point; *arrow* in Fig. 9). This provides a useful means for discrimination of the adhesive forces observed for each sample, comparison of the range of distances at which rupture occurs. Fig. 8*B* displays the adhesion data categorized by the distance at which they occurred and shows that the modal values in this case are different for each sample (140 nm for the wild type sample and 35 nm for the Δ*epsE* mutant). This suggests that the adhesion of the functionalized tip to the wild type sample represents specific interactions with the galactose residues of EPS-2. Validation of the lectin-functionalized tip binding to extracted EPS from the wild type and the Δ*epsE* deletion mutant (both covalently attached to glass slides) confirmed that the PA1 lectin bound only to EPS from the wild type. The frequency of binding was reduced in the presence of free galactose, confirming that it was due to lectin-carbohydrate association (Fig. 8*C*).

Fig. 9 shows three example force spectra obtained on the wild type sample that exhibit well resolved specific adhesive interactions on the retract (*blue*) portion of the force *versus* distance curves that occur well beyond the tip-bacterial surface detachment point. These can be fitted to a wormlike chain polymer scaling model (24, 25) to derive two principal characteristic parameters, persistence length, *L*_p_, and contour length, *L*_c_. Persistence length is a measure of the flexibility of the polymer chain, and contour length provides a direct measure of the molecular size.

## DISCUSSION

The capsular EPS is thought to be involved in the functional properties of colonization and persistence of both commensal and pathogenic bacteria (26, 27). In pathogens, the production of a capsule can be a major virulence factor, yet many of the biosynthetic mechanisms for EPS production are similar between pathogens and commensals. There are few reports on the structure determination and identification of biosynthetic mechanisms of capsular EPS produced by commensal gut bacteria, such as *L. johnsonii* FI9785. In this study, we determined the structure of two different EPS produced *in situ* by this bacterium. We assessed the effects on EPS resulting from the deletion of the *epsE* gene (predicted to encode a UDP-phosphate galactose phosphotransferase that initiates EPS biosynthesis), a spontaneous mutation in the *epsC* gene (*epsC^D88N^*) (described as a putative tyrosine protein kinase) that has a role in the regulation of EPS biosynthesis, and a mutation where the entire *eps* gene cluster had been removed (9).

It was interesting to find that *L. johnsonii* FI9785 was capable of producing two different types of capsular EPS: EPS-1 and EPS-2. EPS-1 is a novel dextran with the unusual feature that every α-(1,6)-linked Glc*p* backbone residue was substituted at O2 with a terminal α-Glc*p* unit. EPS-2 is a heteropolysaccharide that has a unique hexasaccharide repeating unit composed of four glucose and two galactose residues. To our knowledge, the structures of the two exopolysaccharides are unique among EPS produced by any bacteria. The production of α-glucan with different linkages is quite common for the genus *Lactobacillus*, and glucosyltransferases encoded by genes designated as *gtf* are commonly responsible for the production of these dextran-type exopolysaccharides (28–31). The *L. johnsonii* FI9785 genome does not contain any annotated genes with clear homology to glucansucrases. The production of more than one EPS has also been demonstrated in other lactic acid bacteria; *Lactobacillus plantarum* EP56 expressed two heteropolysaccharides, one cell-bound and one released (32), whereas the two EPS produced by *Leuconostoc pseudomesenteroides* R2 were both linear dextrans with different characteristics (33).

EPS phosphorylation has been shown to affect interactions with the host; phosphate groups associated with EPS from *Lactobacillus delbrueckii* subsp. *bulgaricus* have been shown to be required for lymphocyte activation (34), whereas artificial phosphorylation of a dextran from *Leuconostoc mesenteroides* increased its immunostimulatory potential (35). EPS-1 was found to be partly substituted with the 1-phosphoglycerol moiety. Such substitution increases the net charge of the EPS, which could play an important role as determinant of interactions between cells, with host surfaces and with ions and peptides in the environment (32, 36), as well as modulating EPS packing and permeability. Different degrees of phosphorylation and unique phosphorylation patterns may influence the observed differences in cellular adhesion between the wild type and the Δ*epsE* mutant. We found evidence for partial acetylation of both EPS-1 (at multiple sites) and EPS-2 (at a single site), although we did not establish the precise location of the substituents. *O*-Acetylation of bacterial EPS is frequently reported in both lactic acid bacteria (37–40) and others, including *Klebsiella aerogenes*, *E. coli* O8:K27, and the plant pathogen *Pseudomonas flavescens* (41–43). Acetylation can alter the physical properties of the EPS, giving, for example, increased viscosity in solution. In the context of the gut environment, we speculate that acetylation provides protection of the EPS from many types of hydrolases produced by gut bacteria.

AFM was used to investigate cell surface differences using a d-galactose-specific lectin-functionalized tip. The adhesion maps obtained for the wild type (which produces EPS-1 and EPS-2) and the Δ*epsE* mutant (which only produces EPS-1) reveal a clear difference in the frequency and magnitude of adhesive events captured, showing higher adhesion in the wild type, agreeing with the loss of a galactose-rich EPS in this mutant. In addition to detecting and spatially locating the galactose-bearing EPS-2 on the wild type sample, further analysis of the force spectra yielded information about the physical properties of the polysaccharide. Force spectra obtained on the wild type sample fitted the wormlike chain model (24, 25), indicating that EPS-2 adopts a semiflexible random coil conformation. The fact that this information can be obtained *in situ* without the need to isolate the polysaccharide illustrates the power of AFM to measure important intrinsic properties of bacterial cell surfaces (44).

Recently, Fanning *et al.* (45) showed that the putative priming glycosyltransferase Bbr_0430 was essential for the biosynthesis of EPS in *Bifidobacterium breve* UCC2003. In contrast, we found that the Δ*epsE* mutant was still producing EPS-1; this suggested that the production of EPS-1 could be independent from the *eps* gene cluster of *L. johnsonii* FI9785. But deleting this entire *eps* cluster from the genome of *L. johnsonii* FI9785 resulted in the loss of both EPS-1 and EPS-2 production, suggesting that at least one of the genes in this cluster is required for the production of EPS-1. These results are consistent with previous reports where the deletion of the *eps* gene cluster in *L. johnsonii* NCC533 resulted in an acapsular strain (8). The *eps* gene cluster of *L. johnsonii* FI9785 has a genetic organization similar to those of identified gene clusters for the biosynthesis of capsular or extracellular heteropolysaccharides (45–47). We suggest that this gene cluster, which harbors six putative glycosyltransferase genes, might be responsible for the biosynthesis of heteropolysaccharide EPS-2; in addition, one of these glycosyltransferases may have a bifunctional role to produce the homopolymer EPS-1 (48). Alternatively, a novel gene from the genome of *L. johnsonii* FI9785 may be involved in EPS-1 production in conjunction with a gene(s) in the *eps* cluster. Potentially, the six monosaccharide units in the heteropolysaccharide EPS-2 might be added by each glycosyltransferase to form the long-chain capsular EPS-2 initiated by the priming glycosyltransferase *epsE*. Another gene supporting the role of the *eps* cluster in EPS-2 production is the *glf* gene, which putatively encodes the UDP-galactopyranose mutase (9). This has been predicted to convert UDP-galactopyranose to UDP-galactofuranose in *Lactobacillus rhamnosus* GG (47) and may be responsible for the presence of the galactofuranose residue in the repeating unit structure of EPS-2.

Based on our findings, we propose that EpsE is the first glycosyltransferase responsible for attachment of the first sugar monomer to a lipid carrier because the Δ*epsE* mutant was not able to produce EPS-2. The role of this glycosyltransferase has been demonstrated in both Gram-positive and Gram-negative bacteria (46, 47, 49–51). Previously, it was shown that the inactivation of the priming glycosyltransferase of *L. rhamnosus* GG resulted in the absence of the galactose-rich EPS layer on the cell surface, whereas a glucose-rich polysaccharide was still detectable attached to the cell surface (47). Similarly, it was shown that deletion of the *cpsIaE* gene, which initiates the polysaccharide biosynthesis in streptococci, resulted in a non-capsular phenotype (49). In the current study, we showed that after inactivation of the *epsE* gene, a second capsular EPS that was formed by glucose monomers only was still detectable in *L. johnsonii* FI9785. These results demonstrate the essential role of the *epsE* gene in EPS-2 accumulation on the cell surface of lactobacilli, and further work to investigate the *L. johnsonii* FI9785 EpsE protein may confirm its proposed role as the priming glycosyltransferase and identify the first monosaccharide of the chain.

Our previous work on the *epsC^D88N^* mutant showed that there was an increase in the production of EPS in this strain (9). This mutant could produce both EPS-1 and EPS-2, and the alteration of EPS accumulation level was not related to structural changes in the EPS. The increase in EPS content was possibly due to the production of a higher level of EPS-2 than the wild type, related to the putative role of EpsC in the regulation of EPS-2 biosynthesis (49, 52). The characterization of the role of capsular EPS and investigation of the potential genes for EPS-1 biosynthesis is currently in progress.

The structure of capsular EPS has been shown to have an impact on the immunomodulation, biofilm formation, and colonization properties of producing bacteria (4, 45, 53, 54). In terms of the lifestyle of the poultry gastrointestinal tract-derived commensal *L. johnsonii* FI9785, these two EPS could have a protective effect, improving the survival of the bacteria in the external environment and during transit through the gut. Previously, we have reported that differences in the cell surface-associated EPS caused by mutations in the *eps* cluster affect the adhesion and aggregation properties of *L. johnsonii* FI9785 (9). Both of these characteristics can have an impact upon intra- and interspecies interactions as well as interactions with the host gastrointestinal tract. Here we have detected the cell surface changes after mutations in the *eps* gene cluster using anti-*L. johnsonii* FI9785 antibody responses. Górska and co-workers (7) found that the heteropolysaccharide from *L. johnsonii* 142, isolated from the murine gut, reacted to a whole cell antibody. Interestingly, the Δ*epsE* mutant, which could only produce the α-glucan as a capsular EPS, showed a higher antibody response to the *L. johnsonii* antibody than the wild type, and this increase was intensified in the acapsular Δ*eps*_*cluster* mutant, whereas strains producing higher levels of EPS did not show an increased response. The inability to produce EPS-2 or the EPS-1/EPS-2 mixture as a capsular material at the cell surface may have resulted in the exposure and presentation of cell surface epitopes like surface proteins for antibody binding in Δ*eps*_*cluster* and Δ*epsE* mutants. Another explanation for increased antibody response in Δ*epsE* might be that glucose-containing epitopes could be more antigenic than galactose-containing epitopes, as noted previously (55). Deletion of a gene producing a levan EPS from *Lactobacillus reuteri* prevented the induction of regulatory T cells caused by colonization with the wild type strain (54), whereas EPS-deficient strains of *B. breve* elicited a stronger immune response than the wild type (45). EPS layers in these two examples were shown to have a positive effect on persistence and colonization during *in vivo* studies (45, 54). Our findings suggest that the gastrointestinal colonization and recognition of the wild type *L. johnsonii* FI9785, the Δ*eps*_*cluster* and the Δ*epsE* strains by the immune system would be different because of the described structural differences and imply a further biological role for the EPS in protecting the bacteria against an immune response.

In conclusion, this study has revealed simultaneous synthesis of two novel polysaccharide structures by *L. johnsonii* FI9785. Synthesis of both polymers is dependent on the identified *eps* gene cluster; however, the precise regulation of the biosynthesis of individual EPS has yet to be identified. Further structural functional characterization using the isolated mutants will allow us to elucidate the physiological importance of these cell surface structures in bacterial survival, host colonization, and pathogen exclusion.

## Footnotes

4 The abbreviations used are:

EPS: exopolysaccharide(s)
AFM: atomic force microscopy
TEM: transmission electron microscopy
pN: piconewtons
TOCSY: total correlation spectroscopy
ROESY: rotating frame NOE spectroscopy
HMBC: heteronuclear multiple-bond correlation spectroscopy
HSQC: heteronuclear single quantum correlation
MAS: magic angle spinning.
